# Editorial: Everyday Beliefs About Emotion: Their Role in Subjective Experience, Emotion as an Interpersonal Process, and Emotion Theory

**DOI:** 10.3389/fpsyg.2020.597412

**Published:** 2020-11-04

**Authors:** Manuel F. Gonzalez, Eric A. Walle, Yochi Cohen-Charash, Stephanie A. Shields

**Affiliations:** ^1^Department of Psychology, Baruch College & the Graduate Center, City University of New York, New York, NY, United States; ^2^Department of Psychological Sciences, University of California, Merced, Merced, CA, United States; ^3^Department of Psychology, The Pennsylvania State University, University Park, PA, United States

**Keywords:** emotion, affect, lay beliefs, everyday beliefs, emotional experience, interpersonal processes, emotion theory

People hold various beliefs about emotion, such as what causes an emotion, how and why emotions differ, and what one should do about their own and others' emotions (e.g., Ben-Artzi and Mikulincer, 1996; Ford and Gross, 2019). These lay beliefs vary within and between cultures (e.g., Mesquita and Frijda, 1992; Qu and Telzer, 2017), and are transmitted through language (Lakoff, 2016) and socialization practices (Oatley, 1993). Emotion researchers are similarly embedded within cultural contexts and are not exempt from the influence of lay beliefs. Thus, the exploration of lay beliefs of emotion can provide insight into when and how they influence academics and non-academics alike.

The goal of this special issue is to galvanize research on lay beliefs about emotion. As summarized in Table 1, this research can deepen our understanding of (a) how people experience their emotions, (b) emotion as an interpersonal process, (c) the formulation of emotion theory and research practices, and, in turn, (d) open new and exciting avenues for theory and research (see Table 2).

**Table 1:** How lay beliefs about emotion permeate emotion processes, theory, and research.

**Experience of emotion**	**Interpersonal processes**	**Emotion theory and research practices**
*Lay beliefs influence…* …the value placed on particular emotions, and hence whether people seek out or avoid certain emotions …how people appraise their own emotions, and what additional emotions may occur in an emotion episode …emotional behavior in specific contexts or by specific people, such as through expectations about whether an emotion is appropriate …the long-term consequences of an emotion for the person experiencing it, such as through the ways in which people believe they should regulate the emotion	*Lay beliefs influence…* …the (accurate or inaccurate) perception of emotions in other people …the inferences that people make about others who experiencing or express an emotion, and/or about the emotional context …the ways in which people behave toward others, which can suppress or reinforce certain emotions in specific contexts or from specific groups of people …perceived functions or implications of different emotions within a particular interpersonal context, such as conflict	*Lay beliefs influence…* …confirmation biases, in which researchers gravitate toward research questions and hypotheses that confirm their pre-existing beliefs about an emotion …attentional biases in the types of emotional phenomena researchers focus on, or even believe exist …various aspects of the emotion process (e.g., appraisals, emotion regulation, emotion perception), despite rarely being explicitly discussed in emotion theories …the types of information that researchers can gain regarding poorly-understood social phenomena

*We provide examples here of the influences of lay beliefs about emotion. This table is not intended to be exhaustive, and researchers should continue to investigate additional possible influences of lay beliefs*.

**Table 2:** Lay beliefs about emotion: future directions for theory and research.

**Recommendation #1: Further Understand How Lay Beliefs Influence Emotion Processes**
*Sample Future Directions* How do lay beliefs influence emotional processes over the life span or between generations? How do people react in situations where different lay beliefs conflict with each other (for example, when culture-specific beliefs conflict with role-specific beliefs)? Through what mechanisms do lay beliefs influence emotion processes?
**Recommendation #2: Conduct “Meta-Research” Examining How Lay Beliefs Influence Emotion Theories and Research Practices**
*Sample Future Directions* How do research questions and hypotheses about emotions differ across cultures or academic disciplines? How does culture influence the ways in which researchers discuss emotions? To what degree do researchers focus on studying certain emotions over others? What factors influence this attention?
**Recommendation #3: Promote the Theoretical Understanding of Lay Beliefs**
*Sample Future Directions* What roles do lay beliefs play in existing theories of emotion, such as appraisal theories or theories of emotion regulation? Is there room for a theory dedicated solely to the influences of lay beliefs on various emotion processes? How can researchers best conceptually organize lay beliefs about various aspects of emotions, such as its emotion's functionality or its appropriateness in a given context?

## Lay Beliefs and the Experience of Emotion

Lay beliefs about emotions are developed and reinforced in many ways. They both shape and are constructed by the broader culture (Oatley, 1993), can be embedded into specific roles (Hochschild, 1979), and can vary across situations or entire groups of people (Ford and Gross, 2019). People within the same culture may hold different beliefs about emotions depending on their ethnic community—such as indigenous compared to non-indigenous communities—or depending on their role—such as parents compared to teachers (Halberstadt et al.). Thus, everyday understandings of emotions change over time, situations, and people, in accordance with broader cultural and normative expectations.

Once instilled, lay beliefs about emotions permeate the experience of emotion in several ways. First, beliefs shape how people perceive, interpret, and manifest their emotions, such as by influencing the value placed on particular emotions (Ben-Artzi and Mikulincer, 1996), and whether people seek out or avoid certain emotions (Harmon-Jones et al., 2011). For example, Stearns discusses how contemporary United States culture emphasizes childhood happiness, which was uncommon in the early 1800s. Second, lay beliefs influence how people appraise their own emotions, such as whether one's emotions are appropriate to the situation (Warner and Shields, 2009), which could cause additional emotions to occur. For instance, viewing envy as a sin (Silver and Sabini, 1978) may cause people who feel envy to simultaneously experience shame or embarrassment about being envious. Third, because lay beliefs can be situation- or person-specific, certain beliefs may only influence the experience of emotion within specific contexts or may do so differently for different people. As an example of the latter, Sharman et al. found that individuals' gender, self-ascribed gender roles, and gender role attitudes predicted their behavioral crying responses. Lastly, lay beliefs may have long-term consequences for the person experiencing an emotion, such as by influencing their preferred emotion regulation strategies, and, in turn, how they recover from distressing events (Karnaze and Levine).

## Lay Beliefs and Interpersonal Processes

Lay beliefs about emotions also shape how people perceive and interact with others in emotional contexts. For instance, parents and teachers may rely upon their lay beliefs about emotions to identify (sometimes inaccurately) how children feel in a given situation (Hagan et al.; Reschke and Walle). Even beliefs about one's own emotional abilities, such as the ability to take another's perspective, can influence how accurately one recognizes others' emotions (Israelashvili et al.).

Once an emotion is detected, lay beliefs can shape what people infer about the person expressing the emotion and the emotional context (e.g., Zawadski et al., 2013). For example, van Roeyen et al. found that people who perceived a crier's tears as fake (regardless of whether or not they actually were fake) attributed socially undesirable characteristics to the crier (e.g., lower competence and warmth) and judged the crier less favorably. Furthermore, these inferences can feed back into one's lay beliefs about others' emotions, or the groups that others represent. Albohn and Adams, for instance, found that people tend to detect hedonically-unpleasant emotions in the neutral faces of older adults, relative to younger adults, which may foster different lay beliefs about the emotional dispositions of older and younger adults.

Lay beliefs can also influence behaviors toward others. Cultural expectations influence beliefs about whether certain emotions should be experienced or expressed, either by specific persons, in specific situations, or in general (e.g., Hochschild, 1979; Shields, 2005). People can face significant repercussions depending on whether or not their emotions conform to others' expectations (Cheshin). For instance, men not only tend to feel more comfortable about crying in traditionally masculine contexts, compared to non-masculine contexts, but they also are penalized less by others for crying in these contexts (MacArthur). Lay beliefs may thus become more deeply entrenched through encouraging individuals to behave toward others in accordance with their own beliefs, such as suppressing or emphasizing certain emotions in specific contexts. How lay beliefs influence interpersonal processes often depends on aspects of the context. For example, Kurilla found that the concept of conflict varies across cultures, along with the perceived ways that different emotions contribute to conflict. Thus, researchers should account for the broader situational or cultural context when hypothesizing how lay beliefs influence interpersonal processes.

## Lay Beliefs and Emotion Theory and Research

Researchers also have lay beliefs about emotions, which can shape how they study and theorize about emotions. Lay beliefs influence both confirmation biases and attentional biases. Regarding the former, Lindebaum and Jordan (2014) critiqued researchers for overemphasizing symmetrical effects of emotion, in which hedonically-pleasant emotions predict socially desirable outcomes and hedonically-unpleasant emotions predict socially undesirable outcomes. These symmetries may stem in part from value-judgments of these two classes of emotions as being “good” and “bad,” respectively (Cohen-Charash, 2018), and create confirmation biases in emotion research by neglecting the undesirable consequences of hedonically-pleasant emotions and the desirable consequences of hedonically-unpleasant emotions.

Lay beliefs influence attentional biases in the types of constructs researchers focus on or even believe exist, resulting in the emphasis of certain emotional phenomena over others. For example, researchers have given relatively limited attention to neutral affect, compared to positive and negative affect (Watson et al., 1988), despite evidence that neutral affect can influence thoughts and behavior (Gasper et al.). While such attentional biases may facilitate a deeper understanding of some affective phenomena, they can also create theoretical and empirical blind spots regarding other phenomena.

Although lay beliefs influence various aspects of the emotion process—such as appraisals, intrapersonal and interpersonal emotion regulation, and emotion perception—beliefs are only rarely explicitly discussed in the formulation of emotion theory. Lay beliefs may warrant a more central role in emotion theory, which requires a formal effort by researchers to understand and organize them (see Joshanloo, for an example). Lay beliefs may even serve as a starting point to explore poorly-understood phenomena, as Wild and Bachorowski (this issue) suggest regarding social interaction quality.

## What Lies Ahead?

This special issue seeks to spark increased theory and research on lay beliefs about emotion. We offer three focal directions for future theory and research on this topic (see Table 2). First, researchers should further examine how lay beliefs influence the experience, understanding, and regulation of emotions. The articles in this special issue reveal a constellation of lay beliefs, stemming from various sources (e.g., culture, ethnic group, role) and pertaining to various aspects of an emotion (e.g., its functionality, how it should be regulated). Researchers should examine how these and other beliefs influence emotion processes uniquely and in interaction with one another and with the broader context. We further recommend that researchers examine the processes through which lay beliefs influence emotion, and, in doing so, account for both temporal (e.g., momentary, lifespan, generational) and granular (e.g., individual, family, cultural) levels of analysis.

Second, researchers should recognize their lay beliefs, the influence of these beliefs on their research, and how these beliefs promote or hinder a deeper understanding of emotions. To this end, we advocate for “meta-research” focused on understanding how lay beliefs implicitly and explicitly influence emotion theory and research. Such research could consider various factors (e.g., language, culture, personality) that influence how researchers discuss or hypothesize about specific emotions, as well as the amount of attention given to different emotions. As an example of the latter, meta-analytic research could help reveal what emotions and outcomes researchers emphasize or neglect. Furthermore, because lay beliefs are embedded in language, text analytic approaches (e.g., natural language processing) could aid in examining how researchers from different disciplines and cultures discuss emotions.

Finally, we suggest that the various influences of lay beliefs be more explicitly outlined in existing theories about emotions, such as theories about emotion regulation and emotional development. We also suggest that researchers dedicate theories to the influence of lay beliefs about emotions on various emotion processes. Such theoretical developments will enrich emotion science and serve to better connect academic and everyday conceptualizations of emotion.

## Author Contributions

All authors listed have made a substantial, direct and intellectual contribution to the work, and approved it for publication.

## Conflict of Interest

The authors declare that the research was conducted in the absence of any commercial or financial relationships that could be construed as a potential conflict of interest.
